# Cytotoxic and Apoptotic Activity of the Novel Harmine Derivative ZC-14 in Sf9 Cells

**DOI:** 10.3390/ijms19030811

**Published:** 2018-03-11

**Authors:** Jingjing Zhang, Zhijun Zhang, Benshui Shu, Gaofeng Cui, Guohua Zhong

**Affiliations:** 1 Key Laboratory of Crop Integrated Pest Management in South China, Ministry of Agriculture, South China Agricultural University, Guangzhou 510642, China; zhangjingjing@stu.scau.edu.cn (J.Z.); zhangzhijun198803@163.com (Z.Z.); benshuishu@stu.scau.edu.cn (B.S.); 2015102201@stu.scau.edu.cn (G.C.); 2 Key Laboratory of Natural Pesticide and Chemical Biology, Ministry of Education, South China Agricultural University, Guangzhou 510642, China

**Keywords:** harmine, ZC-14, proliferation inhibition, apoptosis, mitochondria

## Abstract

Harmine, one of the natural β-carboline alkaloids extracted from *Peganum harmala* L., exhibits broad spectrum but limited insecticidal ability against many pests. So there is an urgent need to synthesize novel derivatives with high efficiency. In the present study, a new synthetic compound, [1-(2-naphthyl)-3-(2-thioxo-1,3,4-oxadiazol-5-yl) β-carboline] (ZC-14), showed a strong proliferation inhibition effect against the *Spodoptera frugiperda* Sf9 cell line in a dose-dependent manner. Simultaneously, apoptosis induced by 7.5 μg/mL ZC-14 was confirmed with physiological and biochemical evidence, including typical apoptosis characteristics with shrinkage, apoptotic bodies, nuclear condensation/fragmentation, a clear DNA ladder, and a series of apoptotic rates. In addition, mitochondria were confirmed to be involved in apoptosis induced by ZC-14 accompanied with the loss of mitochondrial membrane potential (Δψm), the release of cytochrome c from mitochondria into the cytosol and increased expression of cleaved-caspase-3. However, harmine could not induce apoptosis at the same concentration. In summary, these data indicated that compound ZC-14 has a higher cytotoxicity than harmine against Sf9 cells. Besides, it exhibited an anti-proliferative effect in Sf9 cells via inducing apoptosis in which the mitochondrial apoptotic pathway plays a crucial role.

## 1. Introduction

As the most important group of plant secondary metabolites, β-carboline alkaloids are abundant in *Peganum harmala* L. which is mainly distributed in northwest China and widely used for various diseases therapies due to its remarkable pharmacological effects, including anti-tumor, anti-hypertensive and anti-inflammatory activities [1,2,3]. The alkaloids are also responsible for plant resistance to insects in plant-insect interaction [4]. These plant allelochemicals could affect insect behavior as attractants, repellents or toxic substances, and regulate growth, development and other physiological processes [5,6].

Harmine, a natural β-carboline alkaloid, is abundant in seeds of *P. harmala* [7]. As a therapeutic medicine, harmine was demonstrated as the main effective compound in *P. harmala* for anti-tumor and other pharmacological effects [8]. The mechanism of harmine for human disease therapy is intricate. Harmine performed anti-tumor function by inhibiting the activity of DNA topoisomerase I and II, intercalating into DNA and interfering with DNA synthesis [9,10]. Besides, harmine was considered to be a potential candidate for Alzheimer’s disease therapy by inhibiting acetylcholinesterase (AChE) and butyrylcholinesterase (BChE) activity [11]. More evidence indicated that it could directly dock into the catalytic active site of AChE [12]. Furthermore, harmine might have effects on other diseases by interacting with γ-aminobutyric acid (GABA) receptor, benzodiazepine receptor, opioid receptor, imidazoline receptor and other targets [13,14,15,16,17]. Harmine also has exhibited the potentiality as a botanical insecticide. It was reported that *Plodia interpunctella* was sensitive to harmine and the larval mortality, development disruption and reduction of α-amylase activity was induced after harmine treatments [6]. In addition, harmine shows insecticidal activity against 4th instar mosquito and mustard aphids (*Lipaphis erysimi*) in a dose-dependent manner [18]. Harmine also exhibited toxicity against *Tribolium castaneum* and *Rhyzopertha dominica*; but this was lower than the methanolic extract of *P. harmala* [19].

Harmine has also been used as a lead compound for chemical structural transformation due to broad activity and simple tricyclic structure, and some increasing active derivatives have been synthesized. Simultaneously, the cell lines in vitro are usually the first choice for a preliminary understanding of the efficacy and activity of harmine and its derivatives [20]. Anti-proliferation and apoptosis induction were demonstrated as the main cytotoxic effects. For example, harmine and its analogues have the function of anti-proliferation and apoptosis induction in many cancer cell lines, such as human thyroid cancer TPC1 cells, human colorectal carcinoma SW620 cells and human prostate cancer C4-2 cells [21,22,23]. Our previous research found that harmine could induce G2/M cell cycle arrest and apoptosis in the *Spodoptera litura* SL-1 cell line [24]. Additionally, many apoptosis signaling pathways including the mitochondrial pathway, Fas/FasL-mediated pathways and PI3K/AKT signaling pathways were confirmed to be activated [25,26,27].

For the purpose of obtaining compounds with good insecticidal activity, a series of harmine derivatives were synthesized in our laboratory and the *Spodoptera frugiperda* Sf9 cell line was used for cytotoxicity screening. In this study, the cytotoxicity of harmine and a new harmine derivative, ZC-14, was analyzed. Simultaneously, the physiological and biochemical aspects of apoptosis induced by ZC-14 were evaluated. These results indicated that the cytotoxicity of ZC-14 was higher than harmine and it could induce apoptosis in Sf9 cells through activating mitochondrial apoptotic pathway.

## 2. Results

### 2.1. Harmine and ZC-14 Inhibited the Proliferation of Sf9 Cells

**Figure 1:**
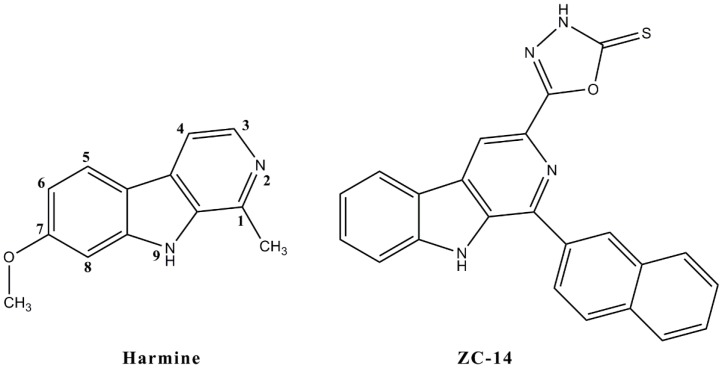
Chemical structure of harmine and its derivative, ZC-14.

**Figure 2:**
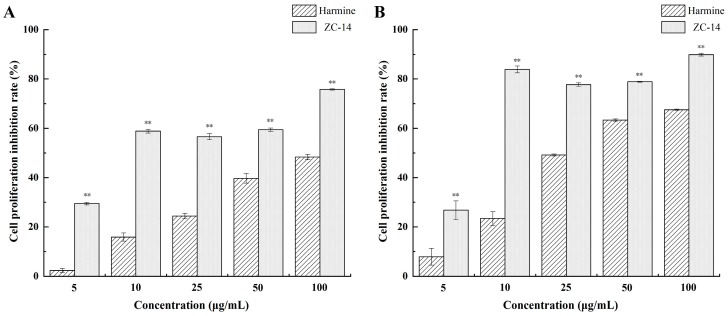
MTT assay of harmine and ZC-14 cytotoxicity on Sf9 cells for 24 h (**A**) and 48 h (**B**). All data were expressed as mean ± SD of three independent experiments. Student’s *t* test was used to compare the groups of each concentration and ** represents *p* < 0.01.

The structures of harmine and a novel harmine derivative, ZC-14, are shown in Figure 1. To evaluate the cytotoxicity of harmine and ZC-14, Sf9 cells were exposed to different concentrations of harmine and ZC-14 for 24 and 48 h. As shown in Figure 2, both of harmine and ZC-14 had the effect of proliferation inhibition. The proliferation inhibition rates of 5, 10, 25, 50 and 100 µg/mL harmine treatments ranged from 2.35 ± 0.85% to 48.36 ± 1.11% while 29.48 ± 0.54% to 75.77 ± 0.26% for ZC-14 after 24 h treatment (Figure 2A). For the 48 h treatment, the proliferation inhibition rates of 5, 10, 25, 50 and 100 µg/mL harmine and ZC-14 ranged from 7.91 ± 3.41% to 67.54 ± 0.23% and 26.80 ± 3.77% to 89.85 ± 0.56%, respectively (Figure 2B). The half maximal effective concentration (EC_50_) values of harmine and ZC-14 against Sf9 cells were determined as 0.469 and 0.0356 mM for 24 h, 0.165 and 0.0146 mM in the 48-h experiment, respectively. These results indicated that harmine and ZC-14 inhibited cell viability in a dose-dependent manner and the cytotoxicity of ZC-14 was higher than harmine in Sf9 cells.

### 2.2. ZC-14 Induced Morphological Changes in Sf9 Cells

**Figure 3:**
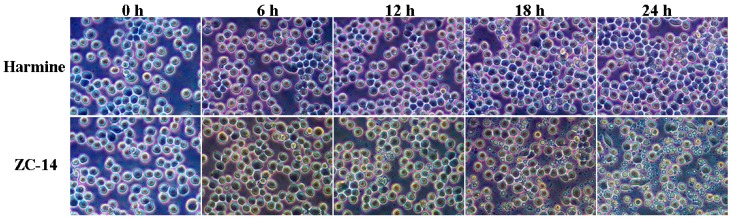
The morphological changes of Sf9 cells treated with 7.5 μg/mL harmine and ZC-14 by inverted phase contrast microscopy (20×).

As shown in Figure 3, the morphological changes of Sf9 cells treated with 7.5 µg/mL ZC-14 for different times were observed under an inverted phase contract microscope. Compared with control cells (0 h), there was no significant morphological change after treatment with 7.5 µg/mL harmine, the cells looked brighter and exhibited a round shape. However, shrinkage and a few apoptotic bodies appeared after ZC-14 treatment for 12 h, although most of the cells had a normal shape. The apoptotic bodies were increased with the prolongation of treatment time. The viable cells were significantly reduced and apoptotic bodies distributed widely for 24 h treatment. These results suggested that ZC-14 with the concentration of 7.5 µg/mL could induce typical morphological characteristics of apoptosis in Sf9 cells, but harmine had no effect on apoptosis induction at the same dosage.

### 2.3. Nuclear Condensation/Fragmentation Appeared in Sf9 Cells after ZC-14 Treatments

**Figure 4:**
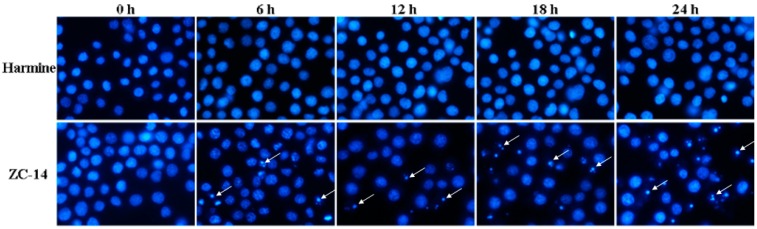
Sf9 cells treated with 7.5 μg/mL harmine and ZC-14 were stained with Hoechst 33258 and the nuclear morphology was observed under a fluorescence microscopy (20×). The nuclear condensation was indicated by the arrows.

Hoechst 33258 is a nucleic acid dye and could be used to visualize the condensation/fragmentation of the nucleus clearly with a blue color. As shown in Figure 4, the control (0 h) and harmine-treatment cells showed normal nuclei with uniform blue color. However, after treatment with 7.5 µg/mL ZC-14 for 12 h, the cells had condensed apoptotic nuclei. These results indicated that 7.5 µg/mL ZC-14 induced nuclear condensation/fragmentation in Sf9 cells, while harmine had no effect on nuclei at the same dosage.

### 2.4. ZC-14 Treatments Induced DNA Fragmentation in Sf9 Cells

**Figure 5:**
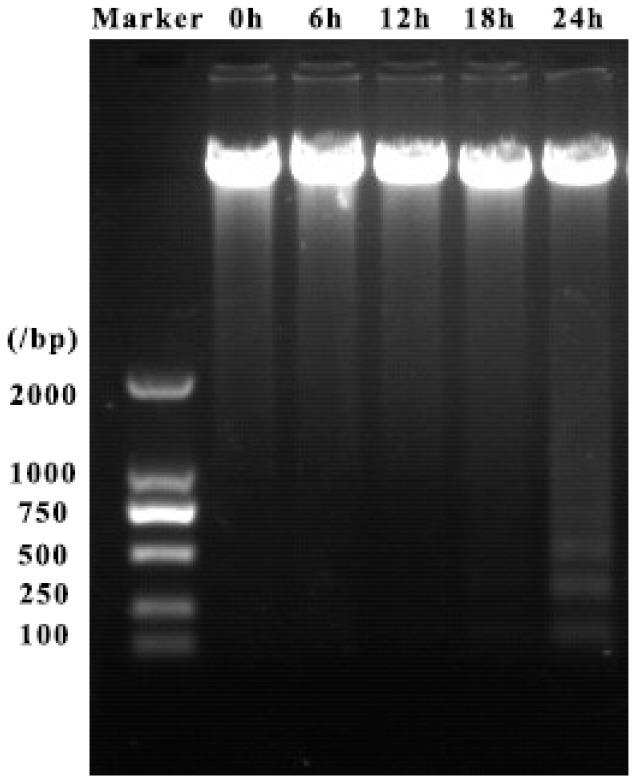
Agarose gel electrophoresis analysis of Sf9 cells’ genomic DNA with different treatments. The cells were treated with 7.5 μg/mL ZC-14 for 0, 6, 12, 18 and 24 h, respectively.

DNA fragmentation is a typical characteristic of apoptosis and DNA samples extracted from cells after treatment with 7.5 µg/mL harmine and ZC-14 were detected by agarose gel electrophoresis. As shown in Figure 5, 7.5 µg/mL ZC-14 treatment for 24 h could induce obvious DNA ladder formation in Sf9 cells. While there was no DNA fragmentation in control cells (0 h) and harmine-treated cells (Figure S1). These results indicated that 7.5 µg/mL ZC-14 could induce a clear DNA ladder in Sf9 cells.

### 2.5. Apoptotic Rate Induced by ZC-14 Treatments in Sf9 Cells

**Figure 6:**
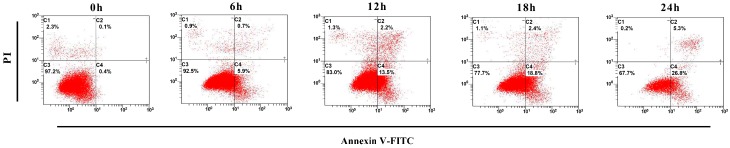
Flow cytometry detected apoptotic rate of Sf9 cells with Annexin V-FITC/PI double staining. The cells were treated with 7.5 μg/mL ZC-14 for 0, 6, 12, 18 and 24 h, respectively.

Annexin V-FITC/PI double staining assays were performed to identify early apoptotic cells and apoptotic rate induced by ZC-14 treatments. According to the results, apoptosis ratio of 7.5 µg/mL ZC-14 treatments was increased gradually in a time-dependent manner. The apoptotic rates induced by ZC-14 in 6, 12, 18 and 24 h were 6.6%, 15.7%, 21.2% and 32.1%, respectively (Figure 6). However, there was no significant difference between control (0 h) and 24 h harmine-treated cells (Figure S2). These above results revealed that 7.5 µg/mL ZC-14 had the effect to induce apoptosis in Sf9 cells and the apoptotic rate increased in a time-dependent manner.

### 2.6. ZC-14 Treatments Increased the Caspase-3 Like Activity in Sf9 Cells

**Figure 7:**
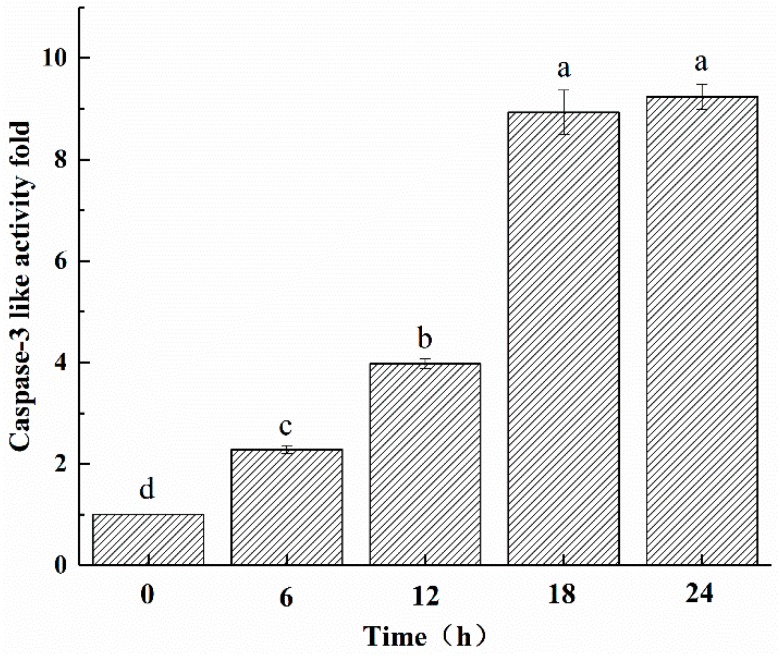
Caspase-3-like activity analysis of Sf9 cells with 7.5 μg/mL ZC-14 treatment for 0, 6, 12, 18 and 24 h, respectively. All data were expressed as mean ± SD of three independent experiments. Different letters above bars indicate significant differences between different treatments (*p* < 0.05) using ANOVA, followed by Duncan’s test.

In this present study, Ac-DEVD-pNA was used as the catalytic substrate for caspase-3-like activity. Increases of caspase-3-like activity were observed after 7.5 µg/mL ZC-14 treatments. After 24 h treatment, caspase-3-like activity reached a maximum value and increased to approximately 9.2-fold compared to control cells (0 h) (Figure 7). These results suggested that ZC-14 could induce apoptosis by activating the caspase cascade.

### 2.7. Loss of Mitochondrial Membrane Potential (MMP) Induced by ZC-14 Treatments in Sf9 Cells

**Figure 8:**
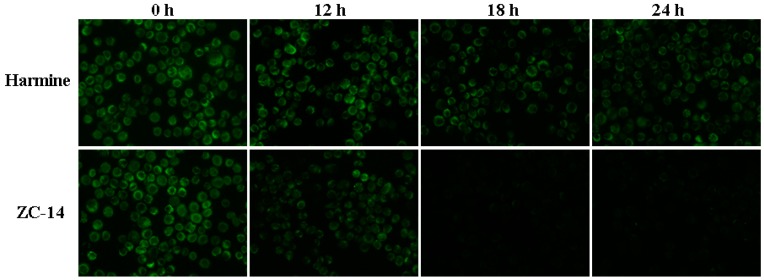
Mitochondrial membrane potential (MMP) changes of Sf9 cells were detected by Rhodamine 123 staining. The cells were incubated with 7.5 μg/mL harmine and ZC-14 for 12, 18 and 24 h, respectively. The treated cells on glass coverslips were stained and the fluorescence of rhodamine 123 with green color was observed by fluorescence microscopy (20×).

To determine whether mitochondria changed after ZC-14 treatment, MMP (Δψm) was detected by Rhodamine 123. As shown in Figure 8, the control cells (0 h) emitted strong green fluorescence and the fluorescence intensity of cells with 7.5 µg/mL harmine treatment were decreased slightly. In contrast, the fluorescence intensity decreased sharply after 7.5 µg/mL ZC-14 treatment in a time-dependent manner. The green fluorescence almost disappeared after ZC-14 treatment for 18 and 24 h. These results indicated that ZC-14 took effect on mitochondria through disruption of mitochondrial membrane potential in Sf9 cells.

### 2.8. ZC-14 Treatments Activated Apoptotic Proteins in Sf9 Cells

**Figure 9:**
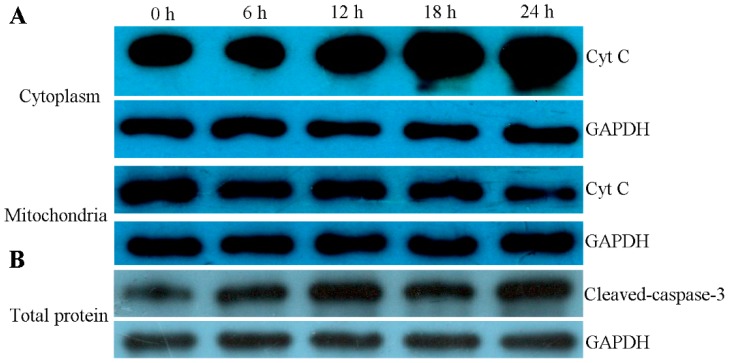
Translational changes of cytochrome c (Cyt C) and cleaved-caspase-3 detected by Western blot analysis. The cells were treated with 7.5 μg/mL ZC-14 for 6, 12, 18 and 24 h, respectively. (**A**) The levels of cytochrome c in cytosol and mitochondria. (**B**) The levels of cleaved-caspase-3. GAPDH was used as an internal control.

In order to confirm whether the mitochondrial apoptotic pathway was involved in ZC-14-induced apoptosis in Sf9 cells, the distribution of cytochrome c in the cytoplasm and mitochondria, and the expression of cleavage-caspase-3 were examined by Western blotting. As shown in Figure 9A, cytochrome c in the cytoplasm was increased, while a significant decrease appeared in mitochondria from 12 to 24 h treatments. Simultaneously, the expression of cleavage-caspase-3 was increased after 7.5 µg/mL ZC-14 treatments (Figure 9B). The results indicated that cytochrome c was released from the mitochondria to the cytoplasm and caspase activation was induced by ZC-14 in Sf9 cells.

## 3. Discussion

*P. harmala* has been used as a traditional Chinese medicine for digestive system neoplasms. A representative β-carboline alkaloid from *P. harmala*, harmine, has been used as an active ingredient for cancer therapy by inhibiting growth and proliferation of various carcinoma cells [28,29], whereas no significant effects have been observed on quiescent fibroblasts [30]. The anti-angiogenic activity of harmine was also reported in B16F-10 melanoma cells of C57BL/6 mice [31]. Methanolic extracts of *P. harmala* had a significant diuretic activity in Wistar albino rats. However, whether harmine was responsible for the effects remains unclear [32]. Previous research showed that dual specificity tyrosine-phosphorylation-regulated kinase 1A (DYRK1A) was one of targets of harmine and the anticancer and antiviral effects were via inhibiting the phosphorylation of DYRK1A substrates [22]. Simultaneously, the adverse effects including deleterious cardiovascular effects and nigrostriatal neurotoxicity, were caused by chronic use of harmine in users and rat. More studies were needed to improve and confirm the availability of harmine for disease therapy [33]. As a newly synthesized harmine derivative, ZC-14 was confirmed to be highly toxic to the insect Sf9 cells, but since in vitro assays are limited, further experimental verification was needed to evaluate whether it could be used as an insecticide for pest control in vivo. Besides, the toxicity of ZC-14 in mammalian or human cells also should be considered and further investigated.

Recently, the structural modification, structure–activity relationships and mechanisms of action as potential agents for cancer therapy have become a hot research topic of harmine. The reasonable transformation of harmine, such as the introduction of appropriate reactive functional groups could significantly improve cytotoxicity. For example, the *N*^2^-benzylated β-carboline derivative compound **3c** has higher cytotoxicity against BGC-823, A375, and KB cell lines with half maximal inhibitory concentration (IC_50_) value of 0.46, 0.68 and 0.93 μM, respectively [26]. In addition, some harmine derivatives synthesized by click chemistry possess predominant cytotoxic effects in cancer cell lines. HBL-100 cells were sensitive to m-fluoro phenyl-1,2,3-triazolyl harmine and the IC_50_ value was 16 μM, less than 32 μM of harmine [34]. A series of novel harmine derivatives transformed at 1 and 3 substitutes were synthesized and their cytotoxicity against human cancer cell lines were detected by MTT; some potential compounds exhibited higher inhibition activities than harmine [35,36]. The 2-thioxo-1,3,4-oxadiazole derivatives with special biological activities have attracted more and more attention and have been widely used in drug and pesticide molecular design in recent years [37,38,39,40]. Our previous study demonstrated that β-carboline 1,3,4-oxadiazoles which introduced the 2-thioxo-1,3,4-oxadiazole groups into β-carboline scaffolds at the C-3 position also exhibited significant cytotoxicity against *Spodoptera frugiperda* Sf9 cells [41]. In the present study, harmine and the novel ZC-14 bearing substituents in positions 1 and 3 of the β-carboline ring have been evaluated insecticides against Sf9 cells. ZC-14 represented higher cytotoxicity with an EC_50_ value of 0.0356 mM for 24 h and 0.0146 mM for 48 h, far less than 0.469 and 0.165 mM of harmine. These results indicated that positions 1 and 3 of the β-carboline nucleus could be important transformation sites and the introduction of suitable substituents could enhance the cytotoxic activities of harmine.

Apoptosis is an autonomous physiological process involved in development, homeostasis and cellular defense of multicellular organism by eliminating unwanted cells [42,43]. Recently, the ability of apoptosis induction in Sf9 cells by many active substances, including azadirachtin, camptothecin, spinosad and abamectin, were investigated and the mechanisms were explored comprehensively with broad studies which were focused on mitochondrial and lysosomal apoptotic pathways [44,45,46,47,48,49]. Previous studies elaborated that harmine also could induce apoptosis in cancer cell lines, but not with the concentration of 7.5 μg/mL in this present study. We speculated that harmine could inhibit the proliferation of Sf9 cells in other modes. In contrast, the representative features of apoptosis, including shrinkage, apoptotic bodies, and nuclear condensation/fragmentation were induced by 7.5 μg/mL ZC-14. These results revealed that 7.5 μg/mL ZC-14 exerted the anti-proliferative effects by apoptosis induction in Sf9 cells.

Mitochondria are the center of intrinsic apoptosis pathways and some pro-apoptotic factors like IBM1 were recruited to mitochondria for the amplification of apoptotic signal [50,51]. In lepidopteran insect cells, the loss of mitochondrial membrane potential (Δψm) was considered to be one of the hallmarks of apoptosis, which led to the increase of outer mitochondrial membrane permeability, and then followed by the release of cytochrome c from the mitochondria to the cytosol, activating caspases and apoptotic signaling pathways downstream [52,53,54]. The releasing of cytochrome c also could be achieved through the pores formed by the large channels constituted with the oligomerization of Bax and Bak [37]. The activation of caspase cascades depended on the apoptosome which was composed of apaf-1 and cytochrome c in the cytoplasm and the response to cellular protein cleavage, and eventually resulted in occurrence of apoptosis [55,56]. However, the role of mitochondria in *Drosophila* apoptosis is not clear and evidence has proven that cytochrome c was not involved in caspases activation [57,58]. In this study, we found that the loss of mitochondrial membrane potential, the releasing of cytochrome c and the increasing expression of cleaved-caspase-3 took place in cells treated with 7.5 μg/mL of ZC-14. These results revealed that the mitochondrial apoptotic pathway could function in apoptosis induced by ZC-14 in Sf9 cells.

## 4. Materials and Methods

### 4.1. Chemicals

Harmine was purchased from Sigma (Santa clara, CA, USA) and the derivative ZC-14 [1-(2-naphthyl)-3-(2-thioxo-1,3,4-oxadiazol-5-yl) β-carboline] was synthesized in our lab (Figure 1). ZC-14 was synthesized using harmine as the lead compound and position 1 replaced with naphthalen and position 3 replaced by 2-thioxo-1,3,4-oxadiazole group [41]. Dimethyl sulfoxide (DMSO) of analytical reagent purchased from Sigma was used as the solvent for dissolving harmine and ZC-14. Rabbit polyclonal anti-cleaved caspase-3 and mouse polyclonal anti-Cyt C was purchased from Cell Signaling Technology (Beverly, MA, USA) and Beyotime Biotechnology (Nanjing, China), respectively.

### 4.2. Cell Culture and Reagents

*Spodoptera frugiperda* Sf9 cells were cultivated in Grace’s insect medium (GIBCO, Grand Island, NY, USA) supplemented with 10% fetal bovine serum (FBS) (GIBCO, Grand Island, NY, USA), 0.33% yeast extract and 0.33% lactalbumin hydrolysate at 28 °C. Cells were changed medium every 3 days and subcultured after 80–90% confluence.

### 4.3. Cell Proliferation Assay

Sf9 cells were seeded into 96-well plates (1 × 10^4^/well) and incubated at 28 °C overnight, then treated with harmine and ZC-14 by the concentrations of 5, 10, 25, 50 and 100 μg/mL. After treatment for 24 or 48 h, 50 μL of 1 mg/mL MTT solution (3-(4,5-dimethylthiazole-2yl)-2,5-diphenyl) was added in each well and the plates were incubated at 28 °C in the darkness for 4 h. The supernatants were carefully discarded and 150 μL of DMSO was added to dissolve the formazan crystal. The absorbance at the wavelength of 490 nm was measured by the Multiskan FC microplate reader (Thermo Scientific, Waltham, MA, USA).

### 4.4. Cell Morphological Observation

Cells with the density of 0.5 × 10^5^–1 × 10^5^ cells/mL were incubated in 6-well plates for 24 h and treated with 7.5 μg/mL ZC-14 for 6, 12, 18 and 24 h, respectively. Morphological changes of cells at different times were recorded by inverted phase contrast microscope (Olympus, Tokyo, Japan).

### 4.5. Hoechst 33258 Staining Analysis

Cells were seeded in 12-well plates and exposed to 7.5 μg/mL harmine and ZC-14 for 6, 12, 18 and 24 h. The supernatant was removed after treatments and cells were fixed with stain-fixative at room temperature for 5 min and washed with phosphate buffered saline (PBS) for two times. The fixed cells were then stained with 500 μL Hoechst 33258 solution (#C1018, Beyotime, Nanjing, China) at 37 °C for 15 min and washed with PBS twice. The nuclear morphology was observed under a fluorescence microscope (Nikon ECLIPES 80 *i*, Tokyo, Japan).

### 4.6. DNA Fragmentation Assay

Cells were seeded into a 6-well plate (0.5–1 × 10^6^/well) and incubated at 28 °C overnight. Harmine and ZC-14 were added into the medium with the final concentration of 7.5 μg/mL. After treated with compounds for 6, 12, 18 and 24 h, DNA was extracted using TIANamp Genomic DNA Kit (#DP304, TIANGEN, Beijing, China) following manufacturer’s protocol. DNA extracted from cells which treated with 1 μg/mL camptothecin for 12 h was used as positive control. 20 μL DNA extractions were subjected to 1% agarose gel electrophoresis with 0.5 µg/mL ethidium bromide at 60 V for 1 h.

### 4.7. Flow Cytometry Assay

Cells were seeded into a 6-well plate (0.5–1 × 10^6^/well) and incubated overnight. Then cells were treated with 7.5 μg/mL ZC-14 for 6, 12, 18 and 24 h and stained by FITC Annexin V apoptosis Detection Kit I (#556547, BD Pharmingen™, Franklin Lakes, NJ, USA) according to manufacturer’s protocol. Briefly, cells with different treatments were harvested by centrifugation (600× *g*, 5 min) and washed twice with ice-cold phosphate buffered saline (PBS). After resuspended with 100 μL 1× binding buffer, cells were stained by 5 μL Annexin V-FITC and 5 μL PI in the darkness at room temperature for 15 min. After that, another 400 μL of 1× binding buffer was added, and the samples were determined by FC500 flow cytometry (Beckman, S. Kraemer Boulevard Brea, CA, USA). The cells with 7.5 μg/mL harmine treatment for 24 h were used as the negative control and 0.5 × 10^5^ cells of each samples were used for analysis.

### 4.8. Caspase-3 Like Activity Assay

Sf9 cells were treated with ZC-14 at 7.5 μg/mL for 6, 12, 18 and 24 h, respectively. After incubation, cells were collected by 600× *g* for 5 min and washed with PBS. Then cells were suspended with lysis buffer (#C1115, Beyotime, Nanjing, China) and incubated in ice for 15 min. After centrifugation at 16,000× *g* at 4 °C for 15 min, the supernatant was transferred to a new tube and the proteins were quantified by Bradford method. For each sample, the supernatant containing 200 µg protein was mixed with 50 μL reaction buffer and 5 μL caspase-3 substrate in a 96-well plate. The plate was incubated in the darkness at 37 °C for 4 h and the absorbance at 405 nm was measured by Multiskan FC microplate reader (Thermo Scientific, Waltham, MA, USA).

### 4.9. Rhodamine 123 Staining

Sf9 cells were seeded on glass coverslips and incubated overnight, then treated with 7.5 μg/mL harmine and ZC-14 for 12, 18 and 24 h, respectively. The supernatant was removed after treatments and cells were stained with Rhodamine 123 (2 mg/mL) (#R8004, Sigma) at room temperature for 20 min. The cells on glass coverslips were washed with cold PBS twice and the fluorescence intensity was detected by fluorescence microscopy (Nikon ECLIPES 80 *i*, Tokyo, Japan).

### 4.10. Western Blot

Sf9 cells treated with 7.5 μg/mL ZC-14 for 6, 12, 18 and 24 h were harvested by centrifugation (600× *g*, 5 min) and washed twice with PBS. Total protein was extracted by CytoBuster^TM^ Protein Extraction Reagent (#71009, Novagen, Kenilworth, NJ, USA) and incubated at 25 °C for 10 min, then centrifuged with 13,000× *g* at 4 °C for 10 min. The supernatants were used as the total proteins samples for western blot. The mitochondrial and cytosolic proteins were isolated by mitochondrial protein extraction kit (#KGP850, KeyGEN, Nanjing, China) according to manufacturer’s protocol. Bradford method was used to determine the protein concentration.

Identical quantities of protein samples were separated by 12% sodium dodecyl sulfate polyacrylamide gel (SDS-PAGE) and transferred to 0.45 μm polyvinylidene difluoride (PVDF) membranes. The membranes were blocked with 5% non-fat milk at room temperature for 2 h and then incubated with primary antibodies diluted 1:2000 in tris-buffered saline (TBS; 100 mM Tris-HCl, pH 7.5, 0.9% NaCl) overnight at 4 °C, followed by secondary antibody conjugated with horseradish peroxidase (diluted 1:4000 in TBS) for 2 h. The protein label was visualized by ECL detection system (Bio-Rad, West Berkeley, CA, USA).

### 4.11. Statistical Analysis

Data were presented as means ± SD (standard deviation) from three independent experiments, and the significance was evaluated by one-way analysis of variance (ANOVA) followed by Duncan’s test and student’s *t* test. *p* value <0.05 was considered statistically significant.

## 5. Conclusions

In summary, ZC-14, the novel compound synthesized based on the structure of harmine, has higher cytotoxicity than harmine. The effect of apoptosis induction by ZC-14 at a lower concentration of 7.5 μg/mL in Sf9 cells was confirmed by physiological and biochemical approaches. The activation of the mitochondrial apoptotic pathway by ZC-14 was reflected by the loss of mitochondrial membrane potential and cytochrome c release. Our results not only confirmed the cytotoxicity mechanism of ZC-14 in Sf9 cells, but also provide some theoretical basis for further use of ZC-14 as a pesticide.

## Supplementary Materials

Supplementary materials can be found at http://www.mdpi.com/1422-0067/19/3/811/s1.
