# Supplementary material for: Cytotoxic and Apoptotic Activity of the Novel Harmine Derivative ZC-14 in Sf9 Cells
Source: Int J Mol Sci. 2018 Mar 11;19(3):811. doi: 10.3390/ijms19030811 (PMC5877672; doi:10.3390/ijms19030811)
Supplement: Supplementary file 1 [file ijms-19-00811-s001.pdf]

International Journal of
Molecular Sciences

Supplementary Materials: Cytotoxic and Apoptotic Activity of the Novel Harmine Derivative ZC-14 in Sf9 Cells

Jingjing Zhang, Zhijun Zhang, Benshui Shu, Gaofeng Cui and Guohua Zhong

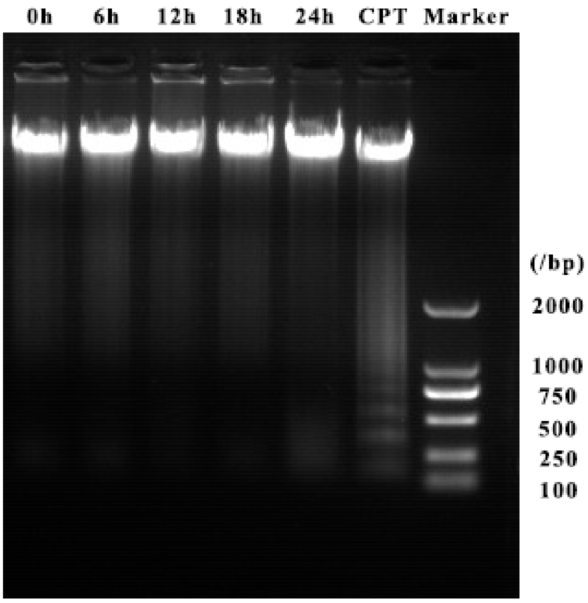

Figure S1. Agarose gel electrophoresis analysis of Sf9 cells genomic DNA with different treatments. The cells were treated with 7.5 μg/mL harmine for 0, 6, 12, 18 and 24 h, respectively. 1.0 μg/mL camptothecin treatment was regarded as positive control.

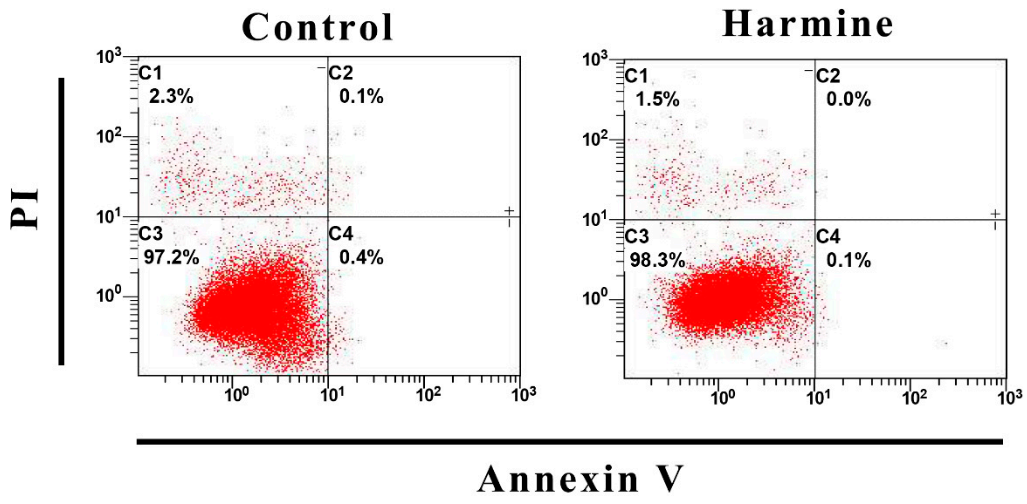

Figure S2. Apoptotic rate of control (0 h) and 7.5 μg/mL harmine treatment cells for 24 h were detected by flow cytometry.
